# Risk Factors Involved in Postural Disorders in Children and Adolescents

**DOI:** 10.3390/life14111463

**Published:** 2024-11-12

**Authors:** Dalia Dop, Vlad Pădureanu, Rodica Pădureanu, Stefan-Adrian Niculescu, Alice Nicoleta Drăgoescu, Aritina Moroșanu, Diana Mateescu, Carmen Elena Niculescu, Iulia Rahela Marcu

**Affiliations:** 1 Department of Pediatrics, University of Medicine and Pharmacy Craiova, 200349 Craiova, Romania; dalia.dop@umfcv.ro (D.D.); aritina.morosanu@umfcv.ro (A.M.); diana.mateescu@umfcv.ro (D.M.); carmen.niculescu@umfcv.ro (C.E.N.); 2 Department of Internal Medicine, University of Medicine and Pharmacy Craiova, 200349 Craiova, Romania; rodica.padureanu@umfcv.ro; 3 Department of Orthopedics, University of Medicine and Pharmacy Craiova, 200349 Craiova, Romania; stefan.niculescu@umfcv.ro; 4 Department of Anesthesiology and Intensive Care, University of Medicine and Pharmacy of Craiova, 200349 Craiova, Romania; alice.dragoescu@umfcv.ro; 5 Department of Medical Rehabilitation, University of Medicine and Pharmacy Craiova, 200349 Craiova, Romania; rahela.marcu@umfcv.ro

**Keywords:** postural disorder, children, risk factors, BMI, physical activity, lifestyle

## Abstract

Postural disorders in children and adolescents have an increasing incidence. The aim of this study was to identify the type of postural defects in school-age and preschool children, as well as the external risk factors determined by an inadequate lifestyle. The research included 134 children aged between 5 and 18 years, in whom postural defects were identified. The project involved an examination of the children’s body posture, a survey of the parents to determine the children’s lifestyle, blood tests, and spinal X-rays. A significant percentage (76%) of the children included in the study were underweight. The examination of postural defects in the students revealed scoliosis in 21% of the patients, kyphosis in 7.5%, and lordosis in 1.5%, while 70% of the patients presented an association between at least two postural defects. As far as risk factors are concerned, we identified the presence of rickets sequelae in 70% of the patients, the presence of pectus excavatum in 43% of the patients, genu varum in 15%, and flat foot in 12%. Additionally, 90% of the children had an incorrect posture at the desk, while 42% incorrectly carried their backpack on one shoulder only. In terms of diet quality, associations between an inadequate diet and postural disorders were found for kyphosis, scoliosis, and other deformities. In conclusion, postural abnormalities in children have an increased incidence from an early age and are a result of the change in lifestyle in recent years, represented by sedentarism, a lack of physical activity, the excessive use of electronic devices, stress, and an inadequate diet.

## 1. Introduction

Characterized as a public health issue, postural changes in children and adolescents have an increasing prevalence, defined by some authors as a social epidemic. While the Pediatric Orthopaedic Society of North America reports the presence of postural disorders in 9.6 million children under 19 years of age [1], studies in Poland report an incidence of between 34 and 69% [1,2], while in China, the prevalence reaches 80% [3], of which 5% are severe deformities.

Although posture is primarily an innate manifestation, it is subject to change and improvement in the process of individual development. Posture is also determined by muscle balance, motor stereotypy, the characteristics of the individual’s higher nervous activity, and character [4].

The causes of postural disorders are divided into congenital and acquired, as well as external and internal. Congenital causes include the various pathologies of intrauterine development, affecting the spine or the lower limbs, while acquired causes are represented by injuries or various pathologies such as rickets, tuberculosis, or the various characteristics of occupational activity (e.g., sitting in a chair with the head leaning forward).

The internal causes of postural disorders are represented by chronic diseases or various hearing and vision defects, which cause the patient to adopt incorrect positions in order to compensate for the deficit [5].

The external causes of poor posture are determined by a person’s lifestyle. In the modern world of computer and electronic development, children choose forms of leisure that involve sitting in front of devices over physical activity [6]. Thus, the muscles in the abdomen and back become weakened over time, leading to a redistribution of the load on the passive structures of the spine and, therefore, contributing to a worsening muscle imbalance and the occurrence of postural disorders and pain in the lumbar spine [7,8].

An important effect on the correct development of the child is rationally organized physical activity during childhood, which contributes to forming and consolidating optimal motor patterns and is a predictor of activity in adulthood [9].

Other causes of acquired postural defects in children are emotional problems, eating disorders, insufficient time for sleep, and the poor organization of work and play spaces—e.g., chairs or tables that are too high, insufficient desk space, etc., which lead to the adoption of wrong postures—and, over time, this static stereotype becomes fixed [10].

Preventing postural defects is a complex procedure aimed at ensuring both physical and mental general health. This issue is particularly important in children and young people because during growth and maturation, posture can be influenced by many factors, and the most important is that the posture developed during this period prevails, to a large extent, throughout life [11,12].

The low effectiveness of measures for the timely diagnosis and correction of postural disorders leads to impaired cardiorespiratory function, impaired metabolism, and irreversible changes in the bone system [13,14,15].

The aim of this study was to identify postural defects among school-age and preschool children, to determine their type, and to identify the external risk factors that are determined by an inappropriate lifestyle or unsatisfactory social conditions related to the occurrence of body posture abnormalities. To this end, we aimed to identify the relationships between the occurrence of postural defects and gender, age, and BMI z-score (WHO), as well as the students’ lifestyle in a broad sense (the extent of physical activity, the duration of homework, and nutrition).

## 2. Materials and Methods

This study involved 134 children aged 5–18 years who were admitted to the pediatric ward or came to the pediatric outpatient clinic and in whom postural defects were identified.

The participating students presented with varying numbers of hours at school or kindergarten, depending on the cycle of education that they were a part of; children were observed to be carrying backpacks of different weights; and school desks were identified as having different sizes. 

### 2.1. Study Design and Data Collection

The research took place between September 2023 and April 2024. The project involved examining the children’s body posture, surveying their parents in order to determine the children’s lifestyle, blood tests, and X-rays of the spine. A data analysis was used to establish the determining factors of postural defects. The parents gave their written consent for the examination of their children and for the questionnaire. Participation in the study was voluntary. 

### 2.2. Measurements

The inclusion criteria were school age, the presence of postural defects, and the obtainment of written parental consent for examination. The exclusion criteria were posttraumatic musculoskeletal disorders, genetic defects affecting the musculoskeletal system, systemic diseases, joint and muscle diseases that were a contraindication for examination, significant vision and hearing impairments, and skin disorders.

The examination of the children consisted of

a. The measurement of the children’s height (in centimeters) and weight. Their BMI (body mass index) was calculated, and the BMI values were classified according to the 2007 WHO (World Health Organization) standards [16]. The z-score and BMI values’ classification into four categories were determined as underweight (a z-score below −2), normal weight (a z-score between −2 and 1), overweight (a z-score above 1), and obese (a z-score above 2).

b. A ruler, a plumb line, and a scoliometer were used to examine body posture.

The examination was performed as follows:

- The child is in a standing position, with their back towards the examiner (who is positioned 1.5–2 m away);
- The positions, the levels, and the inclinations of the different body segments (head, neck, shoulders, arms, and hemithorax) are examined;
- The examination of the landmarks is carried out (posterosuperior iliac spine, iliac crests, scapular spines, shoulder blades, ribs, and vertebral spinous processes), and they are marked with a skin pencil, from bottom to top.

The assessment of specific signs is carried out, taking into account whether the child is right- or left-handed:

- Lumbar bulge, protruding on the convex side of a lumbar curvature (given by the protrusion of the lumbar costiform processes through the muscle mass of the vertebral grooves);
- Rib hump (hemithorax uneveness)—a prominence of the posterior arches of the ribs on the convex side of the thoracic curvature;
- Shoulder asymmetry;
- The unevenness and tilting of the scapular tips (on the convex side, the tip of the scapula is more elevated, more prominent);
- The inequality of the “waist triangles” (between the lateral edges of the thorax and pelvis and the upper limb on that side)—on the concave side, the lower angle of this triangle is sharper and lower than on the opposite side;
- Intergluteal fold—with the cranial extremity towards the convexity of the lumbar curvature;
- Uneven subgluteal folds, lower on side of the convexity of the lumbar spine, the buttock is more prominent.

The examination of the child from the front is carried out, in order to assess the following:

- The degree of symmetry and unevenness of the collarbones, shoulders, and breasts;
- The anterior chondral rib hump (located in the concave part of the main curvature).

Lead wire test—the lead wire is placed at the apex of the C7 spinous process and its position at S1 is observed; if it passes over the S1 spinous process, the scoliosis is balanced; if it passes laterally, the scoliosis is unbalanced on that side; in normal children, the lead wire is placed at the tragus of the ear and it must pass in front of the shoulder and in line with the greater trochanter;

The dynamic examination of the spine is necessary to

- Differentiate a scoliosis proper from a postural defect (scoliotic attitude); the scoliotic attitude is a longer curvature, largely reducible by the anterior flexion of the trunk or the suspension of the child by the head; there are no secondary changes (vertebral rotation); if the curvature persists during the anterior flexion, then the scoliosis is structural.
- Assess, by lateral flexion (lifting the upper limbs from the side of the concavity or association with the lateral inclination of the trunk at 90° towards the convexity), the possibility of the reduction of the lateral inclination and the rotation of the vertebrae of the curvature.

Scoliosis is a deforming condition of the spine, characterized by one or more lateral curvatures visible in the frontal plane, accompanied by the rotation of the vertebrae, with the tendency toward the upper and lower compensation of these curvatures, but without the tendency to reduce them completely (by suspension or decubitus), having an impact on the morphology of the trunk.

Kyphosis is a deforming condition of the spine, characterized by an anterior (sagittal) tilt of the spine, often resulting in a dorsal protrusion, located in the continuity of the spinous processes of the vertebrae.

Hemithorax unevenness (a hump) is a unilateral deformity, seen as a protrusion outside of the spinous processes of the vertebrae, caused by the protrusion of the posterior arches of the ribs and transverse vertebral processes, a consequence of vertebral rotation.

Lordosis is the deforming condition of the spine, rare compared to the previous ones, characterized by a sagittal inclination of the spine, with the concavity oriented posteriorly, or by the accentuation of this curvature that exists physiologically. 

c. For the assessment of muscle strength, we used a Saehan dynamometer. Muscle strength was measured with the children standing upright while squeezing the handle of the dynamometer with full force for 3–5 s. The children had three tries, and the best value was recorded. The results were interpreted according to age.

1. For boys:
  - For ages 8 to 11 years, weights ranged from 13.0 to 18.5 kg;
  - Ages 12 to 15 years—from 21.6 to 37.6 kg;
  - Ages 16 to 19 years—from 45.9 to 51.0 kg.
2. For girls:
  - For ages 8 to 11 years, the standard was from 9.8 to 17.1 kg;
  - For ages 12 to 15 years, the standard was from 19.9 to 28.3;
  - Ages 16 to 19 years—from 31.3 to 33.8 kg.

The second part of the research process consisted of a self-constructed parent/guardian questionnaire, which contained metric questions and detailed questions about the children’s living conditions, eating habits, time spent in front of the computer for study or fun, physical activity, position at their desk, how they wore their backpack, and the length of their study hours.

Subsequently we took blood samples for hemograms and biochemical tests (calcinemia, creatine kinase, alkaline phosphatase, and vitamin D3). In children with scoliosis, we performed X-rays of the spine in order to calculate the Cobb angle. 

### 2.3. Statistical Analysis

The data was processed with IBM SPSS 27. Numerical variables (e.g., age, weight, BMI, hours of classes, hours of play, Cobb angle, etc.) were described as the mean, standard deviation, amplitude and median, as appropriate; to facilitate the risk analysis, for each subject included in the study, the BMI z-score was calculated, with the individual BMI being in relation to the WHO standard values for the age and sex of the subject; categorical variables (e.g., postural deformities, muscle strength, etc.) were presented as absolute and relative frequencies; for normally distributed quantitative variables, the “Independent-Samples T Test” or the ANOVA test were applied; non-parametric tests (the Mann–Whitney U Test or the Kruskal–Wallis test) were used for the asymmetrical distributions; the Pearson Chi Square test or the Fischer’s Exact Test were used to test the association between the risk factors and various postural disorders; the correlation analysis and bivariate logistic analysis were applied and the OR risk was calculated for the various risk factors studied; the results were interpreted for their significance level, assumed at *p* ≤ 0.05.

### 2.4. Ethical Considerations

The research was conducted in accordance with the Declaration of Helsinki and Good Clinical Practice in Research. The Ethics Committee of the University of Medicine and Pharmacy of Craiova, Romania, granted the ethical approval for the study: 244/25 October 2023. Participation was voluntary and the participants were informed about the project.

## 3. Results

1. General characteristics of the study group

A total of 134 children aged 5–18 years participated in the study, of whom 82 (61%) were boys and 52 (39%) were girls, with the majority (61%) of the subjects coming from urban areas.

In terms of their educational level, most of the subjects attended middle school (34%), followed by primary school (33%), and high school (25%); only 6% of the subjects were preschoolers.

**Figure 1:**
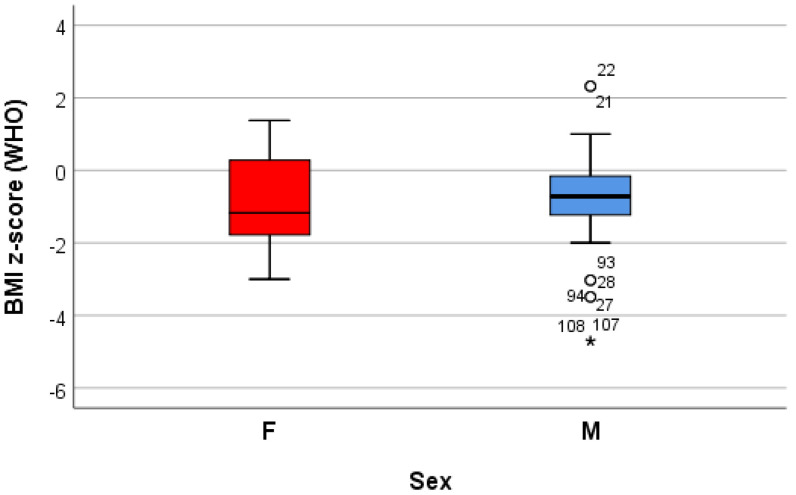
“Box and whiskers” chart representing the BMI z-score (WHO), according to the gender of the patients.

Both the weight and the height of the children varied significantly from a statistical point of view (*p* < 0.05) between the sexes, with girls being thinner and shorter on average than boys; the BMI (kg/m^2^), calculated knowing the height and the weight of the children, varied globally between 10.6 and 23.9, with an average of 16.9 ± 2.7. According to this classification, a significant proportion of patients (76%) were underweight; the values of the standardized BMI score [BMI z-score (WHO)] ranged between −4.7 and 2.3 globally, with an average of −0.8 ± 1.2, values that highlight a batch with small deviations from the normal WHO values established for the sex and age of each child; in relation to the BMI z-score, 83% of the subjects were considered to be of a normal weight status, 13% underweight, 3% overweight, and 1% obese. There were no statistically significant differences in the BMI z-score between the genders (−0.8 ± 1.3 for females and 0.7 ± 01.2 for males, *p* > 0.05) (Table 1) (Figure 1).

2. Postural deformities

The examination of postural defects among students revealed the presence of scoliosis in 21% of the patients, kyphosis in 7.5%, lordosis in 1.5%, while 70% of the patients showed the association of at least two types of defects (Table 2).

For all of the postural defects except scoliosis, the results suggest an association between the postural disorder and the gender component, the cases being statistically significantly more frequent in boys than in girls (*p* < 0.05); only for kyphosis was there an association with the residence environment, the postural disorder being statistically significantly more frequent in urban areas (*p* < 0.05); the frequencies of the other postural deformities were double in the underweight patients compared to the normal weight ones (*p* < 0.05) (Table 3).

The combined effect of the children’s weight and height on the probability of developing a particular postural defect was assessed, based on the values of the standardized BMI score (BMI z-score) and the application of the logistic regression model. The odds ratio value (OR—“event odds ratio”) suggests how many times the chance of a given postural defect in a child increases (when OR > 1) or decreases (when OR < 1) when the BMI z-score increases by 1.

**Figure 2:**
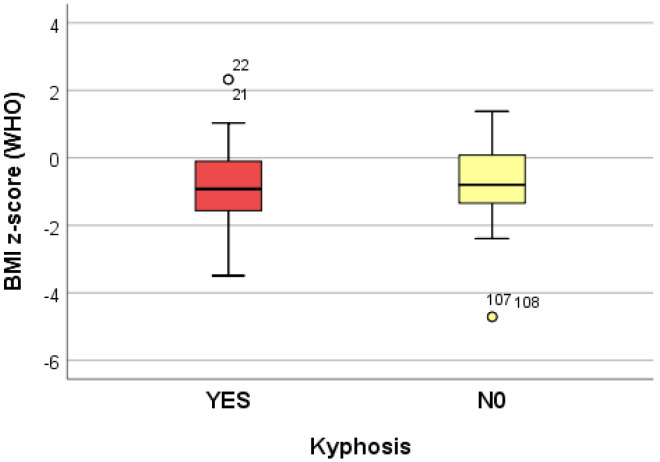
“Box and whiskers” chart representing the BMI z-score (WHO), according to the presence of kyphosis.

**Figure 3:**
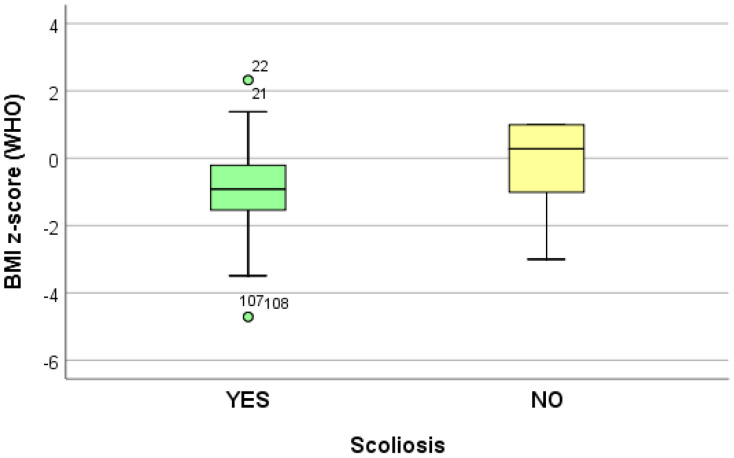
“Box and whiskers” chart representing the BMI z-score (WHO), according to the presence of scoliosis.

**Figure 4:**
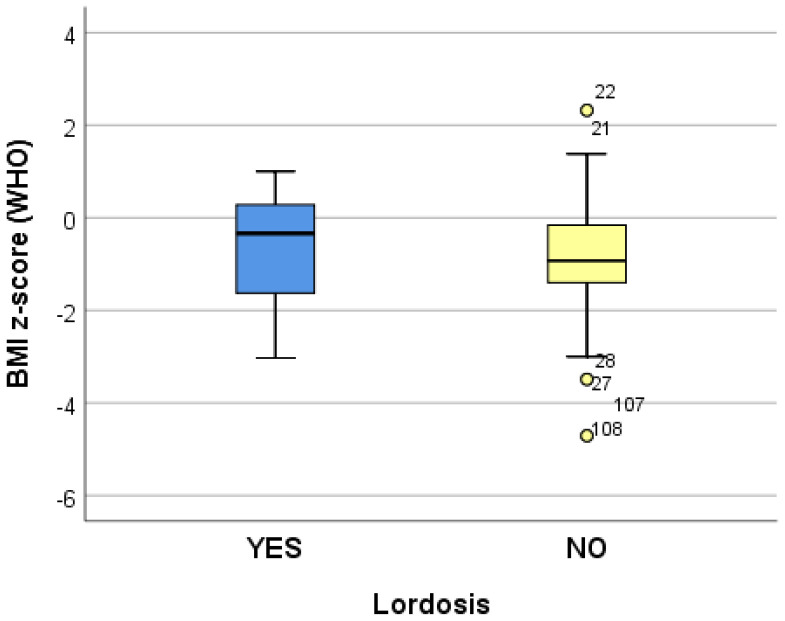
“Box and whiskers” chart representing the BMI z-score (WHO), according to the presence of lordosis.

The data collected from 10 logistic regression models suggest that growing (increasing) the adjusted BMI z-score by one unit reduces the risk of dorsolumbar kyphosis and hemithorax unevenness. Increasing the adjusted BMI z-score by one unit significantly reduces (*p* < 0.05) the risk of developing scoliosis (*p* = 0.039), regardless of the localization; when referring to the localization, increasing the adjusted BMI z-score by one unit significantly reduces (*p* < 0.05) the risk of developing lumbar scoliosis (*p* = 0.020) and increases the risk of developing dorsal scoliosis (*p* = 0.018) (Table 4) (Figure 2, Figure 3 and Figure 4).

For all of the postural deformities, except lordosis, we found statistically significant differences (*p* < 0.05, X^2^ test) between the frequency of the postural deformities and the type of education; the highest significance of the education level was found for hemithorax unevenness and for other postural deformities (Table 5).

The stratified analysis at the level of the associations of the postural deformities also highlighted the influence of the demographic characteristics on the postural defects. There were statistically significant differences (*p* < 0.05) between the sub-batches, defined by the associations of the disorders (Table 6).

We analyzed the factors that may pose a risk to children’s correct posture. These included carrying a backpack on one shoulder, the correct posture at the desk, the time allocated to classes, the time necessary for homework, and the time spent in front of electronic devices (phone, tablet), as well as the physical activity, leisure time, and eating habits of the children. We also looked for the presence of rickets sequelae, flat foot, or congenital club foot, as well as the presence of bone deformities in their parents.

Regarding the risk factors, we identified the presence of rickets sequelae in 70% of the patients, the presence of pectus excavatum in 43% of the patients, pectus carinatum in 27%, genu varum in 15%, flat foot in 12%, and the presence of bone deformities in the parents of 28% of the batch. Additionally, 90% of the children had an incorrect posture at the desk, while 42% incorrectly carried their backpack on one shoulder only (Table 7).

The analysis of the time spent daily in class versus on recreational activities highlights an average of 9 h allocated to classes and homework, 4 h in front of devices, and only 2 h playing (Table 8).

Almost a quarter of the patients (25%, 34 patients) were involved in sports, the most common being dancing (29%, 10 patients), sports involving hands (basketball, fencing, tennis, and volleyball) (29%, 10 patients), soccer (24%, 8 patients), and swimming (6%, 2 patients).

More than half of the investigated patients (69%) declared as their lifestyle an inadequate diet, represented by the predisposition for fast-food or irregular meals; the frequency differed statistically significantly (*p* = 0.000, Fischer’s Exact Test) in relation to the gender component, with boys being the predominant gender group for having an inappropriate diet (83%, 68/82 boys vs. 46%, 24/52 girls).

In terms of diet quality, we found associations between inadequate diet and postural disorders for kyphosis (*p* = 0.001, Fischer’s Exact Test), scoliosis (*p* = 0.001, Fischer’s Exact Test), hemithorax unevenness (*p* = 0.000, Fischer’s Exact Test) and other deformities (*p* = 0.001, Fischer’s Exact Test), as represented in Table 9.

3. The relationship between hand and back muscle strength and posture disorders

In half of the study population (68/134 patients), muscle strength was low (10%, 14/134 patients) or reduced (40%, 54/134 patients). The frequency was significantly (*p* = 0.005) higher in those with kyphosis (60/96 patients with kyphosis vs 8/32 patients without kyphosis) and those with other disorders (*p* = 0.000, 36/46 patients with other deformities vs. 32/88 patients without other deformities), as well as those with hemithorax unevenness (*p* = 0.001, 50/68 patients with hemithorax unevenness vs 18/66 patients without hemithorax unevenness). Statistically significant differences (*p* = 0.024) were also highlighted if the analysis was stratified at the level of the deformity associations (Table 10).

4. The relationship between diagnostic investigations and posture disorders

The stratified analysis at the level of the associations of the postural deformities and diagnostic procedures highlights the effect of vitamin deficiency in the occurrence of associated disorders (statistically significant differences (*p* < 0.05) for all of the assessments (vitamin D3, Ca, Mg, and alkaline phosphatase) (Table 11).

In the global batch of patients with scoliosis, we identified significant (*p* < 0.05) direct, weak (r < 0.4) correlations between the Cobb angle and the demographic characteristics (age, weight, height, and BMI). If localization is taken into account, for patients with lumbar scoliosis we identified significant (*p* < 0.05) direct, medium (0.58 < r < 0.7) correlations between the Cobb angle and the demographic characteristics; for patients with lumbar scoliosis, we identified a significant (*p* < 0.05) direct, weak (r = 0.3) correlation between the Cobb angle and the weight status (BMI) (Table 12).

Testing the correlation between the value of the Cobb angle and the levels of vitamin D, serum calcium, and serum magnesium in the global scoliosis batch revealed statistically significant (*p* < 0.05) indirect, weak (*p* < 0.05) correlations between the Cobb angle and vitamin D3 (r = −0.433), Ca (r = −0.382), and Mg (r = −0.310); by localization, in the case of dorsal scoliosis, we identified significant (*p* < 0.05) indirect, weak-to-medium correlations between the Cobb angle and both Ca deficiency (r = −0.476) and leukocyte alkaline phosphatase (ALP) (r = −0.361); in the case of dorsolumbar scoliosis, we identified significant (*p* < 0.05) indirect, weak-to-medium correlations between the Cobb angle and the levels of Ca (r = −0.478) and Mg (r = −0.439), as well as indirect, intense correlations with vitamin D3 (r = −0.730); we identified direct correlations (r = 0.475) with the ALP level.

In the global scoliosis group, we identified significant (*p* < 0.01) direct, weak correlations with class hours (r = 0.409) and with hours spent doing homework (r = 0.349); between the Cobb angle and the hours spent in front of the screen, the correlation was very weak (r = 0.218); between play hours and the Cobb angle value, we identified significant (*p* < 0.01), indirect, weak-to-medium (r = −0.472) correlations.

In the sub-batches of scoliosis localization, only in the case of dorsal scoliosis was a significant (*p* < 0.05) direct, weak correlation identified between the Cobb angle and the hours spent in front of devices (r = 0.359); in the case of dorsolumbar scoliosis, a significant (*p* < 0.05) direct, medium-to-intense correlation was identified between the Cobb angle and the hours spent learning or doing homework; an indirect, medium correlation was identified with the hours spent playing (r = −0.504) (Table 13).

## 4. Discussion

Postural disorders have an increasing incidence among children, and studies are trying to identify the multiple risk factors involved in their occurrence. The prevention, early diagnosis, and treatment of postural defects, as well as the identification of the modifiable risk factors that may influence the disease and the establishment of appropriate strategies aimed at preventing them, are of great importance [17].

There are certain periods of accelerated growth in a child’s life which are accompanied by postural changes and which require close parental supervision: early childhood and adolescence. Studies indicate that only 18–50% of children and adolescents have a correct body posture [3,18], with the majority of school-age children, especially primary and secondary school children, presenting posture disorders [19].

In our study, the participants were patients aged 5–18 years; most of them were attending secondary school (35%), followed by primary school at 33.5%, but 5.9% were aged 5–6 years, which is in line with the data from previous studies, describing the occurrence of spinal deformities and postural disorders in children in their first year of school (6–7 years) [20,21].

The majority of the children in this study with posture disorders were male, as shown in the study conducted in 2023 by Anna Baranowska [22], while Lei 2020 [3] and Penha [23] indicated a higher incidence in females, which was attributed to lower physical activity in girls compared to boys [24,25].

There are also studies on postural disorders in children that found no statistically significant differences between the parameters characterizing the posture of girls and boys [8,26].

Childhood obesity is a problem of global importance, with the WHO reporting an increase from 8% in 1990 to 20% in 2022. The overloading of the musculoskeletal system, as well as reduced physical activity, leads to the improper development of body posture patterns during the growth and development of children. Numerous studies have shown an increased incidence of postural disorders in overweight or obese children [2,26,27,28], but there are also studies that could not indicate an association between obesity and the occurrence of postural disorders [29,30]. 

In our research, based on the BMI classification (WHO z-score), the group of children examined was mostly in the normal weight range (83%), and no correlation between weight and postural disorders could be established.

The main postural defects found in the study were scoliosis in 86.5% of the patients, followed by kyphosis in 71%, lordosis in 28.4%, flat feet in 12%, and, very importantly, 70% of the children presented with more than two disorders in association.

In Anna Baranowska’s study, the main diagnosis was hyperlordosis (24.1%); scoliotic posture was present in one-third of the children studied, flat back in 18.4%, and the majority (75%) had more than one abnormality [22]. Lazić et al. determined a high incidence of scoliosis (67%) among the children and adolescents studied; hyperkyphosis was present in 27% of the children and hyperlordosis in 6% [31], while Kolarová et al. diagnosed 65% of the children studied with a fallen foot arch, over 30% of the children with spinal curvature deformities in the sagittal plane, and 13% of the children with deformities in the frontal plane [32]. Dragić et al. determined an incidence of 83.9% of the children studied with some type of postural disorder in the sagittal plane [31].

A large number of studies have been conducted on the impact of the weight of a school backpack on children [33,34], the correlation of its weight with gender or age, as well as the effect of asymmetrical carrying on one shoulder only [35,36].

For example, a study carried out by Spiteri et al. in schools in Malta showed that more than 70% of the students who were aged 8–13 years had a backpack exceeding the recommended backpack-weight-to-body-weight ratio of 10%, and a third of the children complained of back pain [37]. In a Polish study, although 60.2% of the students surveyed had a backpack weighing more than 10% of their body weight, no strong, statistically significant correlations were found between the weight of the school backpack and the incidence of postural defects [22].

Carrying a backpack in an asymmetrical manner (on one shoulder) was found to negatively affect the spine, even if the weight of the backpack constituted 10% of the child’s weight, which was previously recommended as a safe shoulder load for a child’s shoulders [38,39].

Similarly, in our study, 42% of the children were carrying the backpack asymmetrically, on one shoulder, but, statistically, it was not possible to correlate asymmetrical carrying with the occurrence of posture disorders.

Another risk factor tracked in our study was poor posture at the home desk or school desk due to the use of non-ergonomic furniture, as was also shown in studies conducted in Poland, where 90% of children had an incorrect posture at the desk [40].

The analysis of time spent in class versus on recreational activities in our study shows an average of 9 h allotted to classes and homework, 4 h in front of devices, and only 2 h for play. Most of the children sit at school desks that are not ergonomically adequate, thus having poor posture.

The list of risk factors associated with posture disorders that were assessed also included habits related to the use of mobile devices (watching TV, using computers, smartphones, and tablets), a common practice, especially among adolescents [41]. Thus, in 2018, approximately 95% of adolescents in the United States had access to smartphones, and, according to an international study, 44.4% of schoolchildren spend 4 h in front of a screen on days without school [42,43].

According to studies, the posture adopted for the use of a smartphone or a computer could cause defects related to the positioning of the shoulder blades in relation to each other and neck and shoulder discomfort, but this topic requires further investigation and new tools to assess the habits of mobile device use among children [44,45].

Nearly a quarter of the patients in our study [25%] engaged in a sports activity, the most common being dancing [29%], sports involving hands (basketball, fencing, tennis, and volleyball) [29%], soccer [24%], and swimming [6%].

In other studies, regular physical activity in school-aged children, at least three times a week, has been shown to help prevent postural abnormalities in the trunk, especially pilates, which can improve rib cage expansion and trunk flexibility [46]. Other sports, such as football and basketball, by improving neuromotor control, provide support to the trunk and pelvis, preventing spinal deformities, while rhythmic gymnastics and ballet are sports that increase the risk of spinal deformities [47,48].

Research conducted in Serbia over the last few decades has shown a linear decrease in the motor abilities of children [24] with postural disorders, correlated with low levels of physical activity [31,49], which was also confirmed in our study, despite the different tests used.

More than half of the patients investigated in this study (69%) reported an inadequate diet as a lifestyle, represented by a predisposition for fast-food or irregular meals, which was also confirmed in Latalski’s study in 2013, in which 59.5% of patients with posture disorders had an unbalanced diet [29].

Testing the correlation between the value of the Cobb angle and the levels of vitamin D, serum calcium, and serum magnesium showed a significant association, which was also confirmed in other preliminary studies that indicated an association between vitamin D and calcium deficiency and a significant increase in the Cobb angle [50], as well as in other studies [51,52].

## 5. Conclusions

The majority of the children in our study showed an association of postural defects, predominantly the boys: they are sitting at school desks that are not ergonomically adequate, carrying their backpacks asymmetrically, spending more hours in front of electronic devices than doing physical activity, and have inadequate diets. Vitamin D, calcium, and magnesium deficiency also accelerates the progression of postural defects.

In conclusion, postural abnormalities in children have an increased incidence from an early age and are a result of negative lifestyle changes in recent years, both in children and their parents, represented by a sedentary lifestyle, a lack of physical activity, the excessive use of electronic devices, stress, and an inadequate diet.

Cohort studies are needed to identify the impact of environmental factors or lifestyle changes that cause the onset of postural disorders in children.

Education, awareness, the early identification of risk factors, and their removal, together with early diagnoses, are effective and imperative.

### Limitations of Study

The limitations of this study are primarily related to the small number of participants and the cross-sectional nature of the study, based on self-reported data, which did not allow us to analyze the cause/effect associations of postural changes and lifestyle, but only the associations between the presence of musculoskeletal changes and the different risk factors through a multivariable analysis.

## Figures and Tables

**Table 1:** Anthropological characteristics of the subjects in relation to the gender component.

Sex		Weight (kg)	Height (cm)	BMI (kg/m^2^)	BMI z-Score (WHO)
F	Normal	52	52	52	52
	Minimum	15.2	96	12.0	−3.0
	Maximum	57.0	185	21.5	1.38
	Median	32.0	144.0	16.2	−1.2
M	Normal	82	82	82	82
	Minimum	13.0	88	10.6	−4.7
	Maximum	62.0	182	23.9	2.3
	Median	49.0	166.0	17.5	−0.7
P		0.007	0.016	0.007	Ns

Legend: F = female, M = male.

**Table 2:** The distribution of the patients in relation to the postural deformities recorded: singularly or with their associations.

Association of Postural Deformities	Patients	
	Absolute Frequency	%
S	28	20.9
K	10	7.5
L	2	1.5
K, L	6	4.5
K, S	24	17.9
K, S, O	30	22.3
K, S, L	14	10.4
K, S, L, O	12	9.0
S, O	4	3.0
S, L	2	1.5
S, L, O	2	1.5
Total	134	100.0

Legend of associations: kyphosis (K), scoliosis (S), lordosis (L), other deformities (O).

**Table 3:** The associations of the postural deformities with the biological and demographic characteristics.

Postural Deformity	Number of Subjects					
	Sex		Background		BMI **	
	F	M	R	U	Normal	Below
SCOLIOSIS	42	74	44	72	30	86
KYPHOSIS	30	66	32	64	22	74
	*p* = 0.004 *		*p* = 0.039 *		*p* = 0.834	
LORDOSIS	20	18	12	26	12	26
	*p* = 0.039 *		*p* = 0.943		*p* = 0.896	
OTHER DEFORMITIES	6	40	18	28	16	30
	*p* = 0.000 *		*p* = 0.988		*p* = 0.032 *	
UNEVENNESS	16	52	28	40	68	50
	*p* = 0.000 *		*p* = 0.873		*p* = 0.997	

* The batches were compared by applying the Pearson Chi Square test; the significance level considered was *p* < 0.05. ** In the case of the analysis that reported the BMI z-score (WHO), the influence of weight status was evident only in the case of kyphosis (*p* = 0.011, X^2^ test). Legend: BMI—body mass index.

**Table 4:** The influence of BMI on the development of postural disorders.

Postural Disorder	OR	OR 95% CI	*p* *	Determination Coefficient (Nagelkerke *R^2^*)
KYPHOSIS	1.022	0.719–1.387	0.889	
Dorsal	1.218	0.730–1.437	0.622	
Dorsolumbar	0.812	0.350–1.428	0.470	
SCOLIOSIS	0.630	0.406–0.977	0.039	0.61
Dorsal	1.538	1.077–2.196	0.018	0.67
Dorsolumbar	0.813	0.599–1.102	0.182	
Lumbar	0.689	0.504–0.943	0.020	0.59
LORDOSIS	1.093	0.803–1.489	0.571	
OTHER POSTURAL DISORDERS	1.046	0.782–1.399	0.762	
HEMITHORAX UNEVENNESS	0.822	0.822- 1.091	0.175	

(*) statistical significance was assumed at *p* < 0.05.

**Table 5:** Distribution of postural deformities by educational level (number of patients).

		Type of Education				*p*
		Kindergarten	Primary School	Middle School	High School	
KYPHOSIS	YES	10	20	38	24	0.020
	NO		24	8	6	
SCOLIOSIS	YES	4	36	42	34	0.006
	NO	6	8	4		
OTHER DEFORMITIES	YES		2	24	20	0.001
	NO	10	42	22	14	
HEMITHORAX UNEVENNESS	YES		4	34	30	0.000
	NO	10	40	12	4	

**Table 6:** The biological characteristics of the patients in relation to the type of association of the postural deformities (including hemithorax unevenness).

Association of Postural Deformities		Age (Years)	Weight (kg)	Height (cm)	BMI (kg/cm^2^)
K	Normal	10	10	10	10
	Median	7.0	24.0	130.0	16.7
K, L	Normal	6	6	6	6
	Median	7.0	21.0	126.0	16.5
K, S	Normal	16	16	16	16
	Median	8.0	26.0	140.5	13.6
K, S, O	Normal	2	2	2	2
	Median	7.0	17.0	116.0	12.6
K, S, O, U	Normal	28	28	28	28
	Median	14.0	49.5	163.0	17.8
K, S, U	Normal	8	8	8	8
	Median	14.5	51.0	171.0	17.4
K, S, L	Normal	10	10	10	10
	Median	8.0	28.0	136.0	16.6
K, S, L, O, U	Normal	12	12	12	12
	Median	16.0	55.5	175.0	17.8
K, S, L, U	Normal	4	4	4	4
	Median	16.5	50.3	174.0	16.6
L	Normal	2	2	2	2
	Median	11.0	42.0	153.0	17.9
S	Normal	20	20	20	20
	Median	9.0	31.5	142.5	15.7
S, O, U	Normal	4	4	4	4
	Median	14.0	44.5	159.00	16.9
S, U	Normal	8	8	8	8
	Median	14.5	46.8	166.00	16.9
S, L, O, U	Normal	2	2	2	2
	Median	17.0	57.0	168.00	20.2
S, L, U	Normal	2	2	2	2
	Median	11.0	27.0	142.00	13.4
P		0.000	0.000	0.000	0.008

Legend of associations: kyphosis (K), scoliosis (S), lordosis (L), other deformities (O), hemithorax unevenness (U).

**Table 7:** The distribution of risk factors in the global batch of postural deformities.

Risk Factors	Patients	
	Frequency	%
Deformities in the parents	38	28
Rickets sequelae	94	70
Flat foot	16	12
Congenital clubfoot	10	8
Hip dysplasia	4	3
Genu varum	20	15
Genu valgum	20	15
Pectus excavatum	58	43
Pectus carinatum	36	27
Desk positions (incorrect)	120	90
Backpack carrying (incorrect)	56	42

**Table 8:** Time allocated to learning and recreational activities, global batch.

	Classes Hours	Device Hours	Homework Hours	Playing Hours
Normal	132	134	120	76
Average	5.5	4.3	3.2	2.1
Median	6.0	4.0	3.0	2.0
Standard deviation	1.2	1.2	0.8	1.1
Minimum	4	2	1	1
Maximum	8	7	5	7

**Table 9:** The distribution of patients with deficient diets in relation to postural deformities and the risk of developing a postural deformity in the case of an inadequate diet.

Postural Deformity	Patients with cu Deficient Diet (n, %)		OR	*p* *
KYPHOSIS (N = 96)	74	77%	3.737	0.001
SCOLIOSIS (N = 116)	86	74%	5.733	0.001
LORDOSIS (N = 38)	24	63%	0.706	0.398
OTHER DEFORMITIES (N = 46)	44	96%	18.33	0.000
HEMITHORAX UNEVENNESS (N = 68)	60	88%	7.69	0.000

(*) statistical significance was assumed at *p* < 0.05.

**Table 10:** The associative distribution of patients with postural disorders in relation to muscle strength in the hands and back.

Postural Deformity	Strength			Total
	Good	Reduced	Low	
K	4	6		10
K, L	4	2		6
K, S	10	4	2	16
K, S, O	2			2
K, S, O, U	6	22		28
K, S, U	4	2	2	8
K, S, L	6	2	2	10
K, S, L, O, U		10	2	12
K, S, L, U		2	2	4
L	2			2
S	20			20
S, O, U	2	2		4
S, U	2	2	4	8
S, L, O, U	2			2
S, L, U	2			2
Total	66	54	14	134

Legend of associations: kyphosis (K), scoliosis (S), lordosis (L), other deformities (O), hemithorax unevenness (U).

**Table 11:** The statistics of the assessments of vitamin D3, Ca, Mg, and alkaline phosphatase in relation to the associations of the postural deformities.

Postural Deformities		Vitamin D3	Alkaline Phosphatase	Ca	Mg
K	Normal	10	10	10	10
	Minimum	21.0	211.0	8.3	1.6
	Maximum	29.0	510.0	8.5	2.2
	Median	22.0	350.0	8.3	2.0
	Mean	23.6	361.0	8.4	2.0
	Standard deviation	3.2	146.0	0.1	0.2
K, L	Normal	6	6	6	5
	Minimum	18.0	170.0	8.1	2.1
	Maximum	25.0	200.0	8.7	2.1
	Median	21.0	200.0	8.6	2.1
	Mean	21.3	190.0	8.5	2.1
	Standard deviation	3.5	17.3	0.3	0.0
K, S	Normal	16	16	16	16
	Minimum	19.0	170.0	8.0	1.7
	Maximum	26.0	470.0	8.7	2.1
	Median	23.0	220.5	8.3	2.0
	Mean	22.8	253.3	8.4	2.0
	Standard deviation	2.2	98.4	0.3	0.1
K, S, O, U	Normal	28	28	28	28
	Minimum	17.0	160.0	8.0	1.8
	Maximum	27.0	518.0	8.6	2.1
	Median	21.0	285.0	8.3	1.8
	Mean	21.3	308.3	8.3	1.9
	Standard deviation	3.1	128.5	0.1	0.1
K, S, U	Normal	8	8	8	8
	Minimum	17.0	150.0	8.1	1.6
	Maximum	25.0	360.0	8.5	2.1
	Median	22.0	164.0	8.2	2.0
	Mean	21.5	209.5	8.3	1.9
	Standard deviation	3.3	100.7	0.2	0.2
K, S, L	Normal	10	10	10	10
	Minimum	19.0	167.0	8.2	1.9
	Maximum	28.0	221.0	8.7	2.1
	Median	22.0	210.0	8.2	1.9
	Mean	23.2	204.4	8.4	1.9
	Standard deviation	3.7	21.4	0.2	0.1
K, S, L, O, U	Normal	12	12	12	12
	Minimum	17.0	150.0	8.1	1.7
	Maximum	22.0	550.0	8.4	1.9
	Median	20.0	275.0	8.2	1.8
	Mean	19.8	333.3	8.2	1.8
	Standard deviation	2.1	163.3	0.1	0.1
K, S, L, U	Normal	4	4	4	4
	Minimum	18.0	205.0	8.0	1.7
	Maximum	20.0	280.0	8.2	2.0
	Median	19.0	242.5	8.1	1.9
	Mean	19.0	242.5	8.1	1.9
	Standard deviation	1.4	53.0	0.1	0.2
S	Normal	20	20	20	20
	Minimum	20.0	170.0	8.0	1.7
	Maximum	31.0	380.0	8.8	2.2
	Median	26.5	207.0	8.5	2.0
	Mean	25.6	239.7	8.5	2.0
	Standard deviation	3.4	70.8	0.3	0.1
S, O, U	Normal	4	4	4	4
	Minimum	21.0	300.0	8.4	1.9
	Maximum	25.0	519.0	8.7	2.0
	Median	23.0	409.5	8.6	2.0
	Mean	23.0	409.5	8.6	2.0
	Standard deviation	2.8	154.9	0.2	0.1
S, U	Normal	8	8	8	8
	Minimum	18.0	210.0	8.3	1.9
	Maximum	22.0	320.0	8.7	2.6
	Median	21.0	215.5	8.5	2.0
	Mean	20.5	240.3	8.5	2.1
	Standard deviation	1.9	53.4	0.2	0.3
	*p*	0.000	0.001	0.000	0.000

Legend of associations: kyphosis (K), scoliosis (S), lordosis (L), other deformities (O), hemithorax unevenness (U). Remark: for the economy of space, we did not include in the table the association groups with less than three patients (C, S, A; L; S, L, O, U; S, L, U).

**Table 12:** Testing the correlation between the Cobb angle and the anthropometric characteristics, scoliosis global batch and by scoliosis localization, respectively.

SCOLIOSIS Global batch		Age	BMI	Weight	Height
	R	0.371	0.310	0.397	0.353
	P	0.004	0.018	0.002	0.007
	N	116	116	116	116
SCOLIOSIS Localization		Age	BMI	Weight	Height
Dorsal	R	0.173	−0.0105	0.051	0.094
	P	0.522	0.698	0.850	0.729
	N	32	32	32	32
Dorsolumbar	R	0.583	0.610	0.676	0.635
	P	0.007	0.004	0.001	0.003
	N	40	40	40	40
Lumbar	R	0.202	0.298	0.283	0.218
	P	0.366	0.049	0.202	0.331
	N	44	44	44	44

Legend: BMI—body mass index.

**Table 13:** Testing the correlation between the Cobb angle and the risk factors represented by the time allocated to various activities; sub-batches—the localization of scoliosis.

Localization of Scoliosis		Classes Hours	Device Hours	Homework Hours	Playing Hours
Dorsal	R	0.129	0.359	−0.0009	0.094
	P	0.633	0.044	0.977	0.797
	N	32	32	28	20
Dorsolumbar	R	0.616	0.285	0.540	−0.504
	P	0.004	0.222	0.000	0.001
	N	40	40	38	20
Lumbar	R	0.223	0.080	0.121	−0.308
	P	0.319	0.724	0.611	0.387
	N	44	44	40	20

## Data Availability

The original contributions presented in the study are included in the article, further inquiries can be directed to the corresponding author.
